# A novel objective for improving the sustainability of water supply system regarding hydrological response

**DOI:** 10.1371/journal.pone.0294578

**Published:** 2023-11-30

**Authors:** Mahdi Moudi, Qiuyan Gai, He Yuan, Li Guiqing, Mahdi Basirialmahjough, Artemis Motamedi, Majid Galoie

**Affiliations:** 1 College of Management, Chengdu University of Information Technology, Chengdu, China; 2 College of Management, Azad University, Mashhad, Iran; 3 Civil Engineering Department, Buein Zahra Technical University, Buein Zahra, Iran; 4 Civil Engineering Department, Imam Khomeini International University, Qazvin, Iran; Arab Academy for Science Technology and Maritime Transport, EGYPT

## Abstract

In general, the sustainability of the water supply system is indicative of an adaptive operational approach, wherein the decision-maker adjusts the system’s performance based on the availability of water resources in a given time frame. In light of this, a novel framework is proposed in this study to evaluate sustainability, including three key indicators: resilience, reliability, and vulnerability. To address stressors that may lead to system failure, a multisectoral water allocation optimization is undertaken. In order to account for the future implications of climate change on the hydrological cycle, a simulation step, is incorporated, utilizing the Soil and Water Assessment Tool (SWAT) under various emission scenarios (RCP4.5 and RCP8.5), prior to integrating the streamflow data into our proposed optimal framework. To calibrate and validate historical data (2014–2019) and simulate future streamflow patterns (2025–2085), the Sistan Basin, located in an arid region of Iran, is analyzed as a case study. In light of the anticipated adverse impacts on the water supply system, certain adaptation measures, such as demand shrinkage scenarios, are considered to further appraise the proposed framework. Based on the final output, it is evident that the agricultural and industrial sectors, being the primary water consumers, are more susceptible to negative impacts resulting from the reduction in system sustainability. This susceptibility is primarily attributed to their highest vulnerability and comparatively lower reliability.

## Introduction

As a consequence of rapid population growth and the impact of climate change, the fulfillment of water demand in a regional water supply system is poised to emerge as a formidable challenge in the foreseeable future [1–3]. The scarcity of available water resources, coupled with inadequate water supply management, progressively jeopardizes the sustainability of the aforementioned water supply system [4]. As climate change reduces water resources, the equilibrium between water demand and supply will be disrupted to such an extent that an insufficient quantity of water will be available to meet the long-term requirements of system participants, thereby resulting in a failure of system performance [5, 6].

It is necessary to highlight that the capacity to uphold system performance amidst failure conditions is defined as a pivotal attribute of system sustainability [7]. In this context, the optimization of the supply-demand imbalance would serve to propel the system towards failure safety, enhance the adaptability and efficacy of performance, and consequently led to improved sustainability [8].

The analytical performance indicators utilized in the management of water resources on a large scale specifically in the context of water supply systems, have been recognized as a means of enhancing sustainability across diverse hydrological conditions [9, 10].

However, in recent times, there has been a growing emphasis on scientific research pertaining to the sustainability of water supply systems, primarily driven by the uncertainties associated with runoff and the profound influence of human activities in basins. For instance, Afshar et al. [11] devised an integrated framework, including both cyclic and non-cyclic components, to address various scenarios of uncertainty and thereby enhance the sustainability of water allocation in irrigation systems. Butler et al. [12] formulated a comprehensive framework, including reliability, resilience, and sustainability, to analyze the challenges faced by water supply systems and their consequential impacts on system performance, with the ultimate goal of improving sustainability. Abdi-Dehkordi et al. [13] proposed a spatial distribution framework, employing a system dynamics approach, to evaluate the behavior of water supply systems in terms of both quantitative and qualitative environmental aspects, thereby enhancing system sustainability. Kotir et al. [14] introduced an integrated dynamic tool to assess the extent of interaction between users and water resources in the agricultural sector of the Volta Basin in West Africa, with the aim of enhancing the sustainable management of water resources in the irrigation system. Chen et al. [15] developed a hierarchical water resources programming model based on a credit-based system, which effectively manages the sustainability of a regional water supply system.

In prior studies, the sustainability of water supply systems has been extensively analyzed as a paramount concern in the context of multisectoral participants, whose influence extends to various economic, social, and environmental factors. Previous studies have primarily put forth one or two indicators derived from hydrological time series, which consider sustainability without taking into account optimal performance and the projected climate patterns. Therefore, these specific measures implemented during periods of system failure often fail to meet the predefined threshold. Hence, the development of a scientific methodology grounded in long-term planning is necessary to bolster water supply strategies and enhance system sustainability. Moreover, the management of a water supply system is complicated due to the presence of multiple stakeholders [3]. In essence, a long-term strategic decision-making process that disregards the availability of water resources is destined for failure, as certain regions experience water surplus while others suffer from unmet water demands.

Considering this objective, this study formulates a comprehensive framework for optimizing water supply with the aim of improving the sustainability of the water supply system under future scenarios. Factoring in the inherent uncertainty associated with climate, the initial data are extracted utilizing the SWAT tool, and the proposed model is subjected to analysis under diverse hydrological responses.

According to the above descriptions, the main contributions of this study are listed below:

1. A novel optimization framework is introduced to tackle the long-term water supply challenge while simultaneously examining the level of system sustainability across various sectors.
2. Considering the impact of climate patterns on water resources, the Sistan Basin, located in an arid region of Iran, is selected as a case study. The SWAT tool is employed to extract the initial data, and subsequently, the developed model is analyzed for the time periods [2025–2055&2056–2085].
3. Due to the inherent uncertainty in the supply process, several adaptation measures, including demand shrinkage, are implemented to evaluate and scrutinize the periodic decisions.

## Materials and methods

### Projecting climate patterns

The first step in projecting climate patterns through SWAT involves the analysis of future climate impact postulation (General Circulation Models (GCMs)). GCMs provide a quantitative analysis of climate impacts at regional scales based on various emission scenarios (RCP4.5&RCP8.5) and hydrological measures. These models effectively incorporate complex interactions in response to greenhouse gas emissions, surface water levels, and climate change simulations [16]. However, due to their large spatial resolution, GCM results are not sufficiently reliable for smaller regional scale features. To address this limitation, a refined downscaling technique (statistical method) is employed [17]. In this regard, downscaled models such as the Hadley Center Coupled Model (HadCM) and the European Center of Medium-Range Weather Forecast (ECMWF), along with different SRES-scenario reports (*A*_1_,*B*,*B*_1_, and *A*_1_), have been developed to investigate climate patterns for future periods (2025–2055&2056–2085). The spatial variability of the basin is then classified into Hydrological Response Units (HRUs), and subsequently, the characteristics of each HRU are analyzed with respect to soil type, topography (DEM), slope classes, and land use.

**Fig 1:**
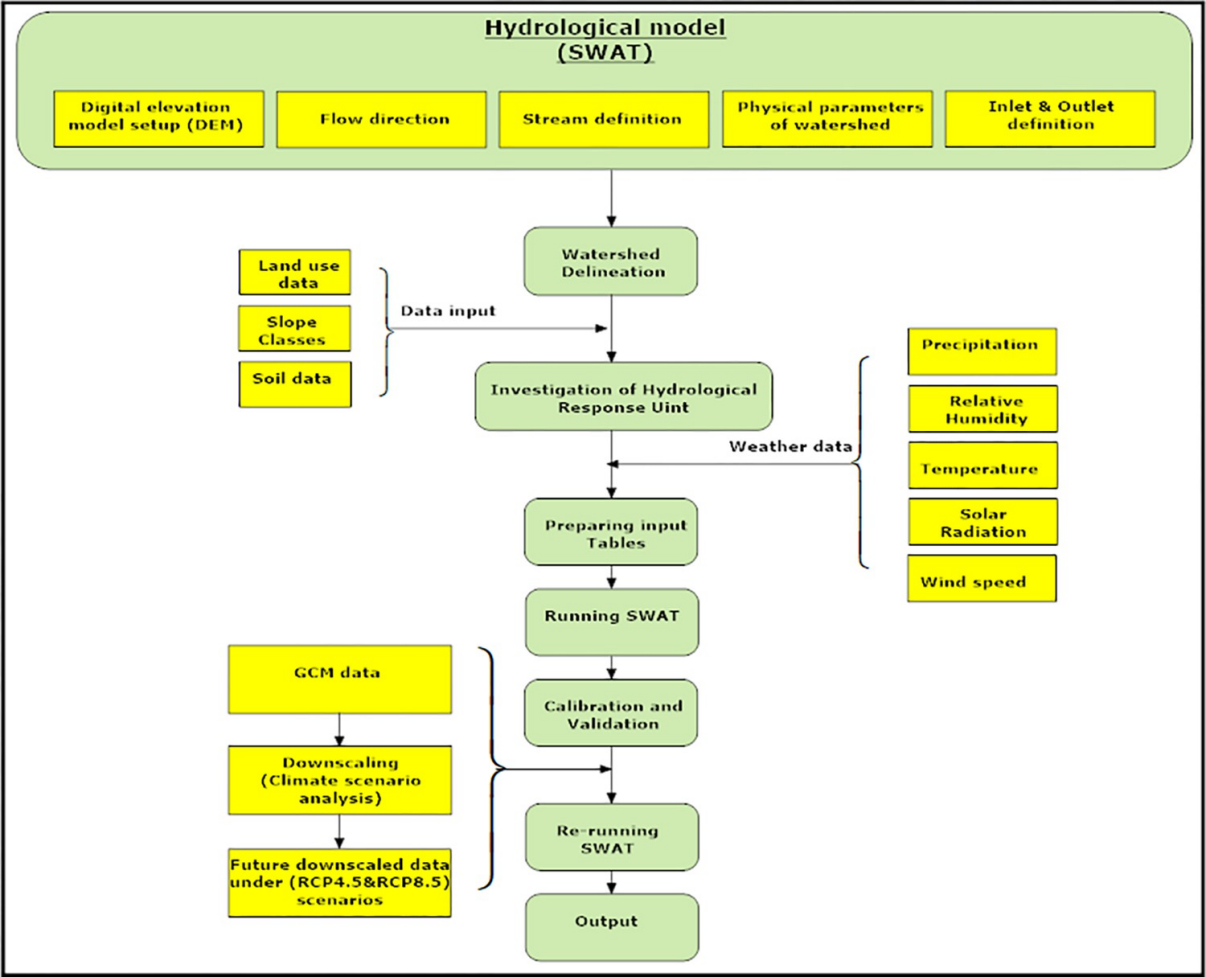
Investigation of hydrological response unit.

The model calibration and validation process utilizes two time periods: 2004–2013 (10 years) and 2014–2019 (6 years). During this process, various statistical measures, including the coefficient of determination *R*^2^, Nash-Sutcliffe efficiency (*NSE*_*rel*_), Nash-Sutcliffe coefficient (*NSE*), and root mean square error (*RSR*), are employed to assess the model’s performance in the region. In addition, sensitivity analysis of parameters is conducted using SWAT-CUP (95% PPU) to ensure that the simulated data aligns closely with the observed data. The output data generated by the SWAT model is subsequently utilized in the proposed sustainability framework (Fig 1).

### Novel sustainability assessment framework

**Fig 2:**
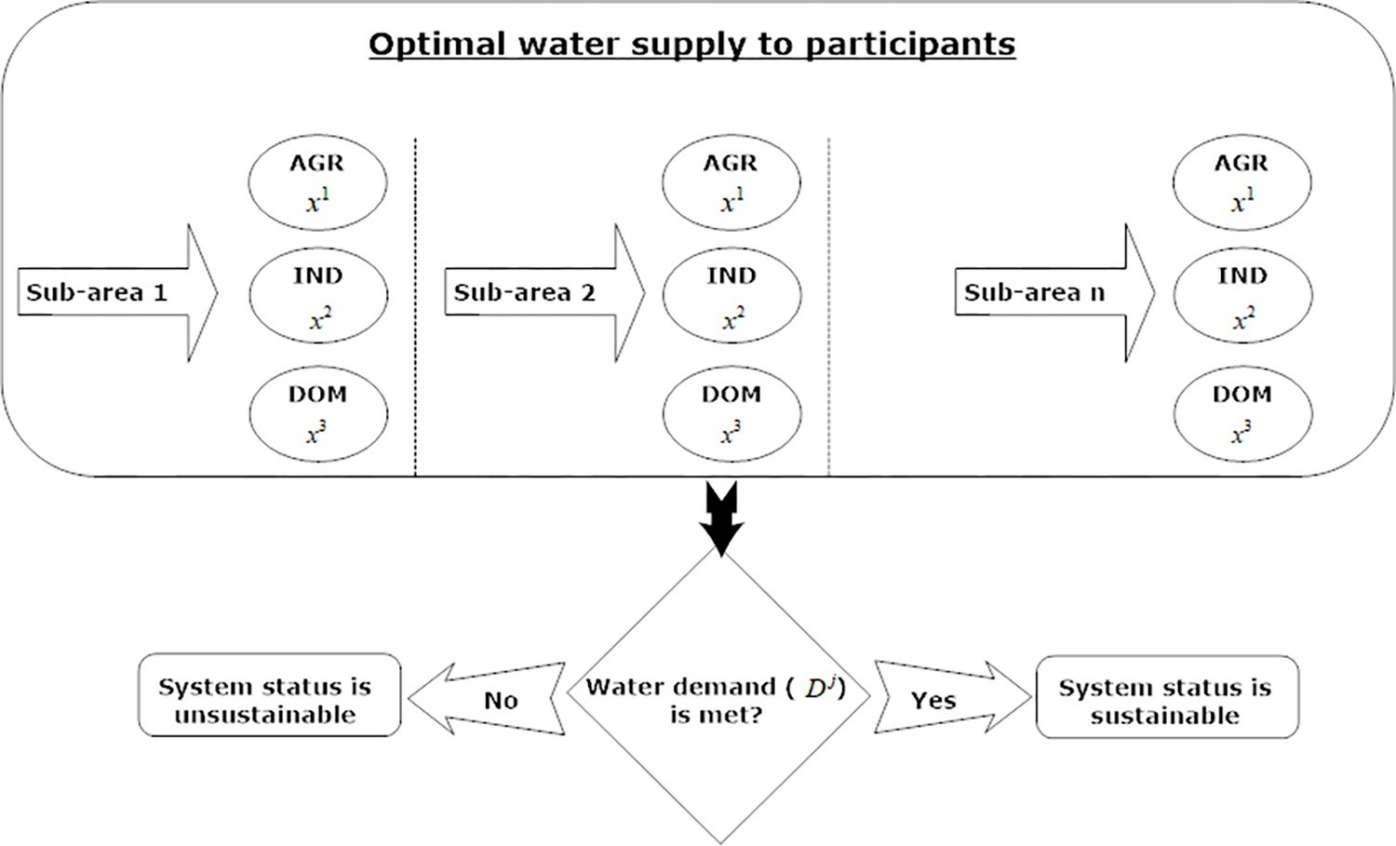
The developed framework fluxogram for improving the sustainability of water supply system.

According to the aforementioned descriptions, the sustainability of the water supply system involves the integration of performance indicators to investigate adaptation measures from diverse perspectives in a specific timeframe [18, 19]. When evaluating the performance of the water supply system, three indicators are taken into account: resilience, vulnerability, and reliability [20, 21]. Resilience refers to the comprehensive understanding of the system’s status and attempt to restore the system during a crisis, thereby mitigating the rate of system failure [22, 23]. Reliability denotes the system’s capacity to meet consumer expectations and address failures by fulfilling water demand [24, 25]. Vulnerability serves as a tool to assess the failure of water crises and its impact on consumer dissatisfaction [26]. In essence, the primary criterion for these indicators is to strike a balance between water demand and supply intervals, thereby reducing the failure rate in the water supply system. As defined by Karamouz et al. [27], these three factors collectively constitute the definition of sustainability measure. Therefore, a model is developed to optimize water supply across various sectors, with the aim of enhancing the sustainability of the water supply system under future scenarios (Fig 2). The proposed model is solved utilizing R software version 4.3.1. Subsequently, the developed model is expounded upon in greater detail.

The definition provided for the sustainability of the water supply system was determined as follows [27]:

$$S_{t}^{j}=\eta_{t}^{j}*\lambda_{t}^{j}*(1-\beta_{t}^{j})$$ (1)

Where, $S_{t}^{j}$ refers to the sustainability index, $\eta_{t}^{j}$ is the reliability index, $\lambda_{t}^{j}$ refers to the resilience indicator, and $\beta_{t}^{j}$ is vulnerability index during period *t* in sector *j*. Indeed, reliability, resilience, and vulnerability are considered as performance indices of water supply system [12].

According to the aforementioned definition, the reliability of the system denoted the probability that the system would persist in a satisfactory state over a specified duration. Therefore, the reliability of the water supply system was assessed based on its adherence to a predetermined criterion of demand fulfillment [21]:

$$\eta_{t}^{j}=\frac{1}{T}\sum_{t-1}^{T}(1-\mu_{t}^{j})\;t=1,\ldots ,T\;and\;j=1,\ldots ,m$$ (2)

Where, *T* is the total period, $\mu_{t}^{j}$ treats as a binary variable (0 and 1), hence if the requested demand is met in period *t*, the system is satisfied and minimum $\mu_{t}^{j}$ = 0, otherwise, minimum $\mu_{t}^{j}$ = 1.

Resilience, on the other hand, refers to the system ability to swiftly recover from a state of failure to a state of non-failure. Therefore, the resilience of a system was quantified as the probability of it not encountering any failure-related issues in a certain timeframe [20]:

$$\lambda_{t}^{j}=\frac{\sum_{t=1}^{T}\rho_{t}^{j}}{T-\sum_{t=1}^{T}\mu_{t}^{j}}$$ (3)

Where $\rho_{t}^{j}$ is defined as the transition between failure and non-failure modes in the given period whose value is 1 or 0:

$$\rho_{t}^{j}=\{\begin{array}{ll} 1 & if\;y_{t-1}^{j}\in failure\;and\;y_{t}^{j}\in non-failure\\ 0 & Others \end{array}$$ (4)

It must be mentioned that $y_{t}^{j}$ is referred to a given time series of a parameter of interest.

The vulnerability of a system, as defined, represented the probability of failures occurring in a specified period [9]. In the context of the water supply system, the vulnerability indicated the severity of the failure state [26].


$$\beta_{t}^{j}=\frac{1}{T_{V}^{j}}\sum_{t=1}^{T}\frac{\mu_{t}^{j}*R_{t}^{j}}{D_{t}^{j}}$$ (5)

Where, $D_{t}^{j}$ and $R_{t}^{j}$ indices represent water demand and water scarcity, respectively. In addition, $T_{V}^{k}$ is the total number of shortage periods as follows:

$$T_{v}^{j}=\sum_{t=1}^{T}\sum_{j=1}^{m}\mu_{t}^{j}$$ (6)

However, the degree of water scarcity in period *t* is as follows:

$$R_{t}=\{\begin{array}{l} \sum_{j=1}^{m}D_{t}^{j}-\sum_{j=1}^{m}x_{t}^{j},\;\sum_{j=1}^{m}D_{t}^{j}\rangle \sum_{j=1}^{m}x_{t}^{j}\\ 0\;,\sum_{j=1}^{m}D_{t}^{j}\langle \sum_{j=1}^{m}x_{t}^{j} \end{array}$$ (7)

However, the rate of water shortage in sector *j* was contingent upon the disparity between water demand $D_{t}^{j}$ and water supply $x_{t}^{j}$ (as a decision variable) in period *t*.

Drawing upon the proposed long-term assessment framework, the primary objective of this study was to maximize the sustainability of the water supply system in the following manner:

$$\underset{x_{t}^{j}}{\max}F=\eta_{t}^{j}*\lambda_{t}^{j}*(1-\beta_{t}^{j})$$ (8)

*The constraints are listed below*:

The dynamic process of determining the volume of water in the reservoir relied upon the volume of available water in the preceding period and the rate of runoff, as depicted in Eq (9). In addition, the allocation of water to different sectors necessitated that the allocated volume be less than the reservoir’s total capacity, as illustrated in Eq (10). Additionally, the volume of water transferred to the sub-areas had to be less than the available water in the reservoirs, as exemplified in Eq (11).


$$l_{t}=\max[l_{t-1}-\sum_{j=1}^{m}x_{t}^{j}+I_{t}-\bar{l},0]$$ (9)

$$0\leq \sum_{j=1}^{m}x_{t}^{j}\leq l_{t}$$ (10)

$$l_{t}^{\min}\leq l_{t}\leq \bar{l}$$ (11)

Where, *l*_*t*−1_ is the volume of available water in the reservoir in period *t*−1, *I*_*t*_ is the rate of runoff during the period *t*, $\bar{l}$ is referred to the maximum capacity of the reservoir.

However, the global framework of this study is as follows:


$$\begin{array}{l} \underset{x_{t}^{j}}{\max}F=\eta_{t}^{j}*\lambda_{t}^{j}*(1-\beta_{t}^{j})\\ \left\{ \begin{array}{l} \eta_{t}^{j}=\frac{1}{T}\sum_{t-1}^{T}(1-\mu_{t}^{j})\;t=1,\ldots ,T\;and\;j=1,\ldots ,m\\ \lambda_{t}^{j}=\frac{\sum_{t=1}^{T}\rho_{t}^{j}}{T-\sum_{t=1}^{T}\mu_{t}^{j}}\\ \beta_{t}^{j}=\frac{1}{T_{V}^{j}}\sum_{t=1}^{T}\begin{array}{l} \frac{\mu_{t}^{j}*R_{t}^{j}}{D_{t}^{j}} \end{array}\\ T_{v}^{j}=\sum_{t=1}^{T}\sum_{j=1}^{m}\mu_{t}^{j}\\ R_{t}=\left\{ \begin{array}{l} \sum_{j=1}^{m}D_{t}^{j}-\sum_{j=1}^{m}x_{t}^{j},\;\sum_{j=1}^{m}D_{t}^{j}\rangle \sum_{j=1}^{m}x_{t}^{j}\\ 0\;,\sum_{j=1}^{m}D_{t}^{j}\langle \sum_{j=1}^{m}x_{t}^{j} \end{array}\right.\\ l_{t}=\max[l_{t-1}-\sum_{j=1}^{m}x_{t}^{j}+I_{t},\bar{l},0]\\ 0\leq \sum_{j=1}^{m}x_{t}^{j}\leq l_{t}\\ l_{t}^{\min}\leq l_{t}\leq \bar{l}\\ \rho_{t}^{j}=\left\{ \begin{array}{l} 1\;if\;y_{t-1}^{j}\in failure\;and\;y_{t}^{j}\in UN\;failure\\ 0\;Others \end{array}\right. \end{array}\right. \end{array}$$

### Case study and data collection

**Fig 3:**
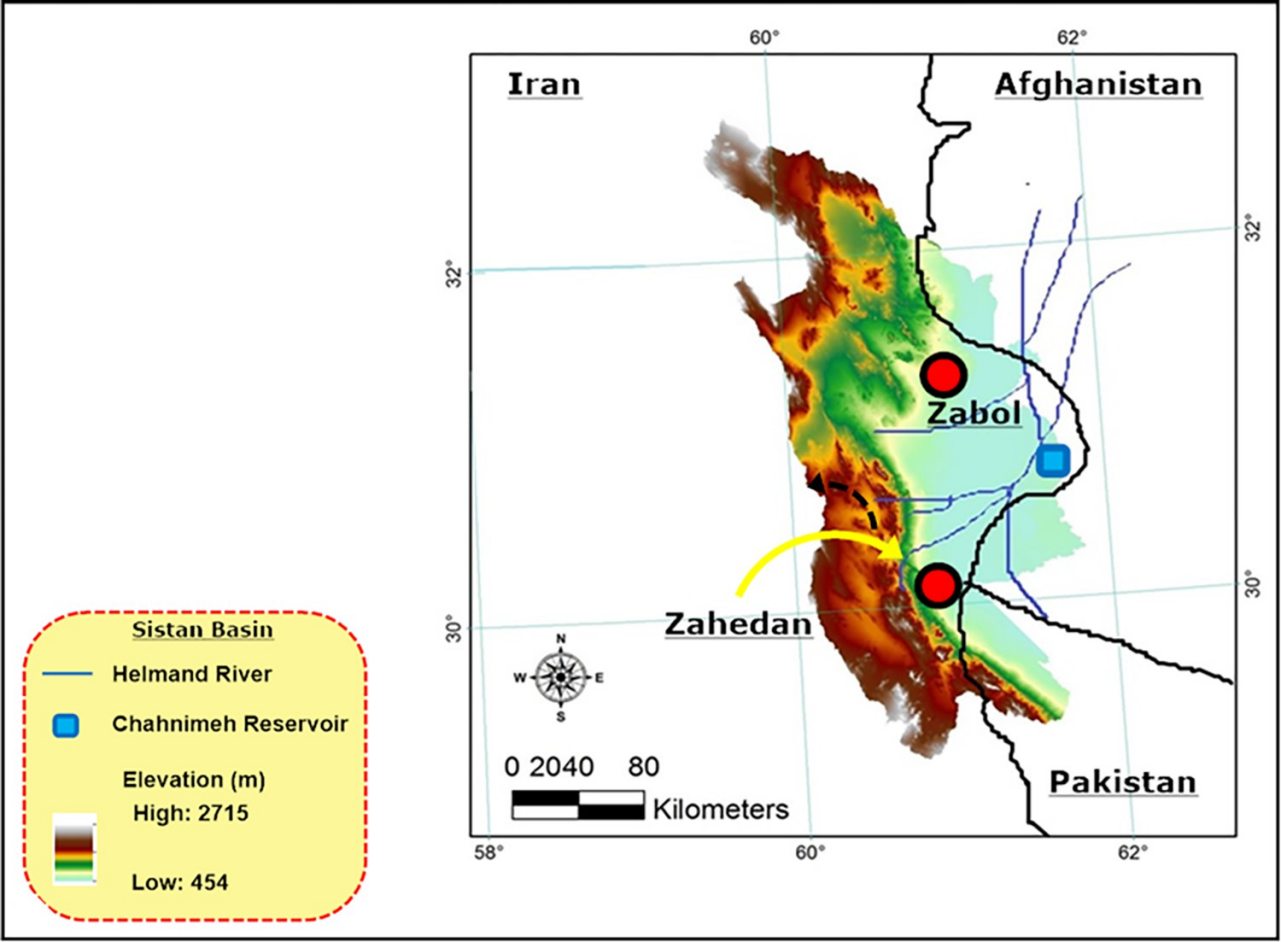
Sistan Basin location, Iran, map created using ESRI software.

The Sistan Basin (30°–31.5° *N to* 61°–66° *E*), also referred to as the Hamoon watershed, is situated in the Sistan and Baluchestan province of Iran, in close proximity to the Iran-Afghanistan border [28]. In recent years, the region has experienced an exceedingly severe drought, primarily attributable to the prevailing climatic conditions and significant decline in the average annual precipitation, which predominantly occurs during the winter season [29]. In addition, the Afghan government has undertaken the construction of several dams along the Helmand River (as a transboundary river) with the intention of impounding runoff. However, this development has exacerbated the drought situation [30, 31]. Thus, in order to meet the water requirements of two sub-areas, namely Zabol and Zahedan, decision-makers have redirected the runoff originating from the Afghanistan side to the Helmand River, utilizing four large reservoirs collectively known as Chahnimeh [32–34]. As the drought intensifies and water resources continue to dwindle, decision-makers have encountered significant challenges in maintaining a balance between water demand and supply across various sectors. Therefore, the Sistan Basin has been identified as the focal area of study (Fig 3).

The basin delineation and DEM of the study area, with a resolution of 30 meters, were derived from the National Aeronautics and Space Administration (NASA). The soil map data were obtained from the site (soilgrids.org). The land use data associated with the study area were extracted from GLC30 (globallandcover.com) dataset, provided by globallandcover.com, with a resolution of 300 meters. The Sistan Basin was further divided into 39 sub-basins based on detailed Hydrologic Response Units (HRUs), which took into account topography, soil type, slope characteristics, and land use. In addition, the observed streamflow data used in this study were obtained from the Regional Water Organization (sbrw.ir) and the Meteorological Organization of Sistan and Baluchestan (sbmeteo.ir). To forecast water demand, two criteria were considered: the correlation rate between Gross Domestic Product (GDP) and population growth. These criteria were used to analyze water demand in the agricultural, industrial, and domestic sectors [35, 36]. The water demand in these sectors, as assessed by the Iranian Water Research Center, (Table 1).

**Table 1:** Parameter of water demand in sub-areas.

j		2017	2030s	2040s	2050s	2060s	2070s	2080s
Zabol (10^6^m^3^)	**AGR**	49.17	52.65	50.82	52.00	55.62	53.31	53.92
	**IND**	11.58	10.86	12.09	13.29	13.01	16.93	14.78
	**DOM**	5.16	6.17	6.64	7.14	6.48	7.86	7.32
	**AGR**	24.38	23.79	21.85	24.07	25.21	27.69	28.41
Zahedan (10^6^m^3^)	**IND**	33.14	35.16	43.79	37.19	41.56	42.27	41.96
	**DOM**	19.43	21.14	22.68	23.36	24.08	23.76	27.50

AGR = Agricultural sector, IND = Industrial sector, DOM = Domestic sector.

2017 is benchmark year.

## Results and discussion

### Downscaling procedure to simulate climate data

For model calibration, the SCS-CN method was employed to estimate monthly precipitation, as it is the most sensitive parameter. To accomplish this, 16 sensitive parameters varying ranks were developed to analyze the inflow rate (Table 2).

**Table 2:** Parameters of sensitivity response to flow rate.

Parameter	Description	Unit/ Fitted rang	Lower bound	Upper bound	Variation
**ALPHA_BF**	Baseflow alpha factor	Day	0	1	Replace
**BLAI**	Maximum potential leaf area index	m^2^ m^-2^	0	1	Replace
**CH-K2**	Effective hydraulic conductivity in the main channel alluvium	mm h^-1^	0	150	Replace
**CN2**	Initial SCS runoff curve number for moisture condition II	[–1,1]	-25%	25%	Replace
**ESCO**	Soil evaporation compensation factor	[–1,1]	0	1	Replace
**GW-REVAP**	Groundwater”revap” coefficient	[–1,1]	-0.036	0.036	Add
**GW-DELAY**	Groundwater delay time	Days	-10	10	Add
**REVAPMIN**	Threshold depth of water in the shallow aquifer required for return flow to occur	mm	-1000	1000	Replace
**Sol-K**	Saturated hydraulic conductivity	mm h^-1^	-25%	25%	Add
**SOL-Z**	Depth from the soil surface to the bottom of layer	mm	-25%	25%	Add
**TLAPS**	Temperature lapse rate	°C km^-1^	0	10	Replace
**BIOMIX**	Biological mixing efficiency	1–1	0	1	Replace
**CANMX**	Maximum canopy storage	Mm	0	10	Add
**CH-N2**	Manning’s “n” value for the main channel	s m^-3^	0	1	Add
**EPCO**	Plant update compensation factor	[–1,1]	0	1	Replace
**GWQMN**	Threshold depth of water in the shallow aquifer required for return to occur	mm	-1000	1000	Add

Variation: means the default parameter is replaced by a given value (Replace); or the existing parameter value is multiplied by 1+ given value (Add)

**Fig 4:**
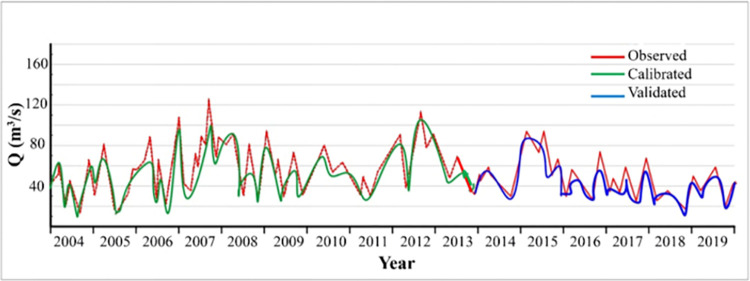
Calibrated and validated monthly flow rate in Sistan basin.

However, the similarity between the calibrated and observed outputs is highlighted through analysis (Table 3 and Fig 4). The *R*^2^ and *NSE* values, as two practical statistical measures, indicate coefficients of acceptability (values exceeding 0.5) and reduced variance error, respectively, thereby signifying a satisfactory level of congruence with the simulated data.

**Table 3:** Statistical analysis for inflow at gauging stations for calibration and validation periods.

Weather Station	Calibration Period (2004–2013)				Validation Period (2014–2019)			
	*R* ^2^	*NSE*	*NSE*_*rel*_	*RSR*	*R* ^2^	*NSE*	*NSE*_*rel*_	*RSR*
**Zabol**	0.78	0.75	0.72	0.63	0.72	0.70	0.81	0.57
**Zahedan**	0.73	0.69	0.68	0.53	0.76	0.83	0.76	0.63
**Nosratabad**	0.77	0.71	0,80	0.56	0.74	0.71	0.88	0.54
**Ladiz**	0.70	0.82	0.78	0.52	0.86	0.91	0.69	0.58

**Fig 5:**
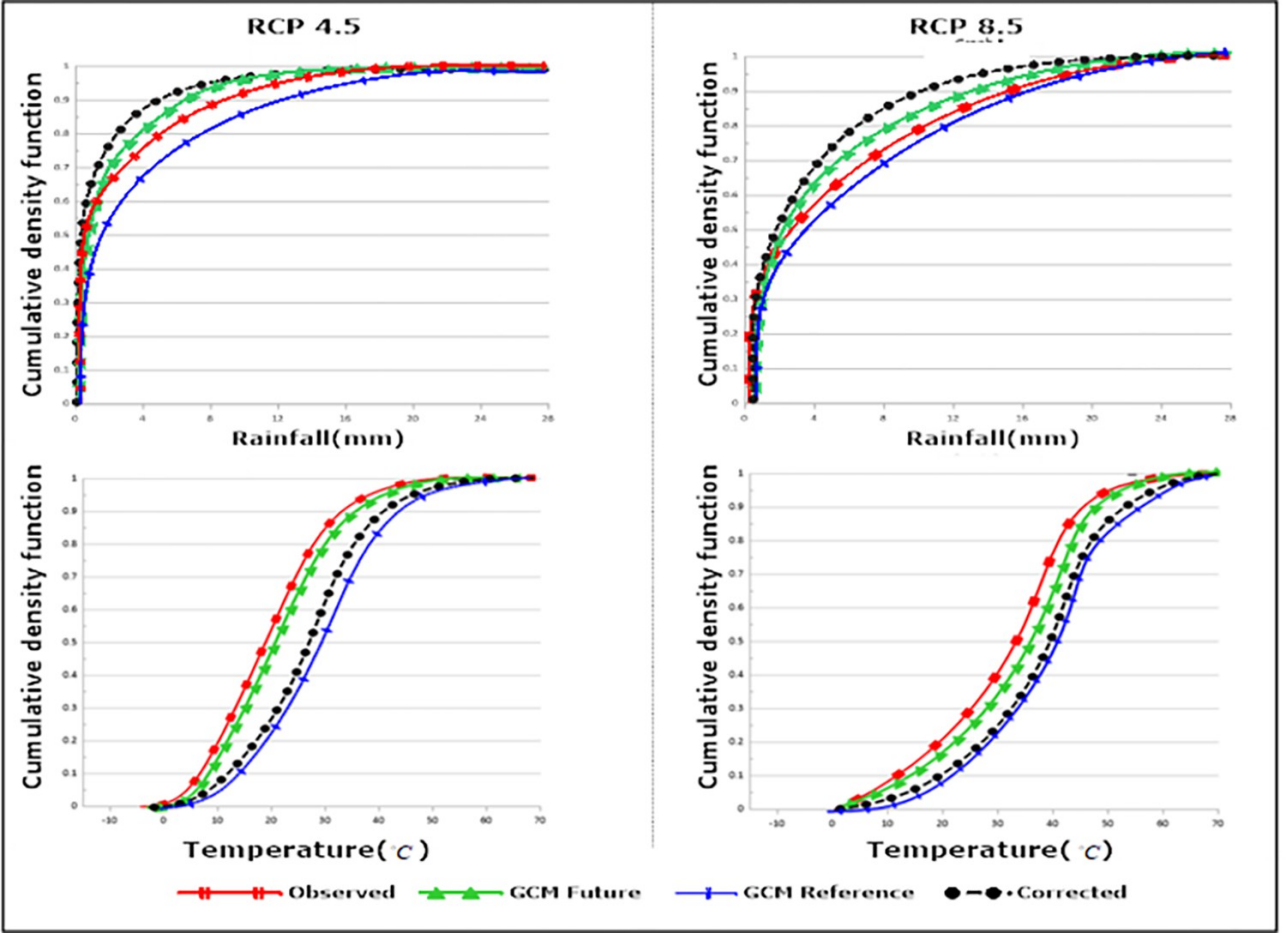
Cumulative distribution functions response to observed and corrected data (2025–2085) under RCP4.5&RCP8.5 scenarios.

Meanwhile, the application of frequency adaptation was employed to rectify the disparity in frequency between the simulated and observed data [30]. Indeed, frequency adaptation is regarded as an investigative tool to explore the empirical frequency discrepancy between projected and observed drought occurrences [37]. Fig 5 illustrates the prognostication of rectified rainfall and temperature under the RCP4.5&RCP8.5 scenarios for the period (2025–2085). In this context, both rainfall and temperature factors are practically overestimated, leading to the negative correction terms for both scenarios.

**Fig 6:**
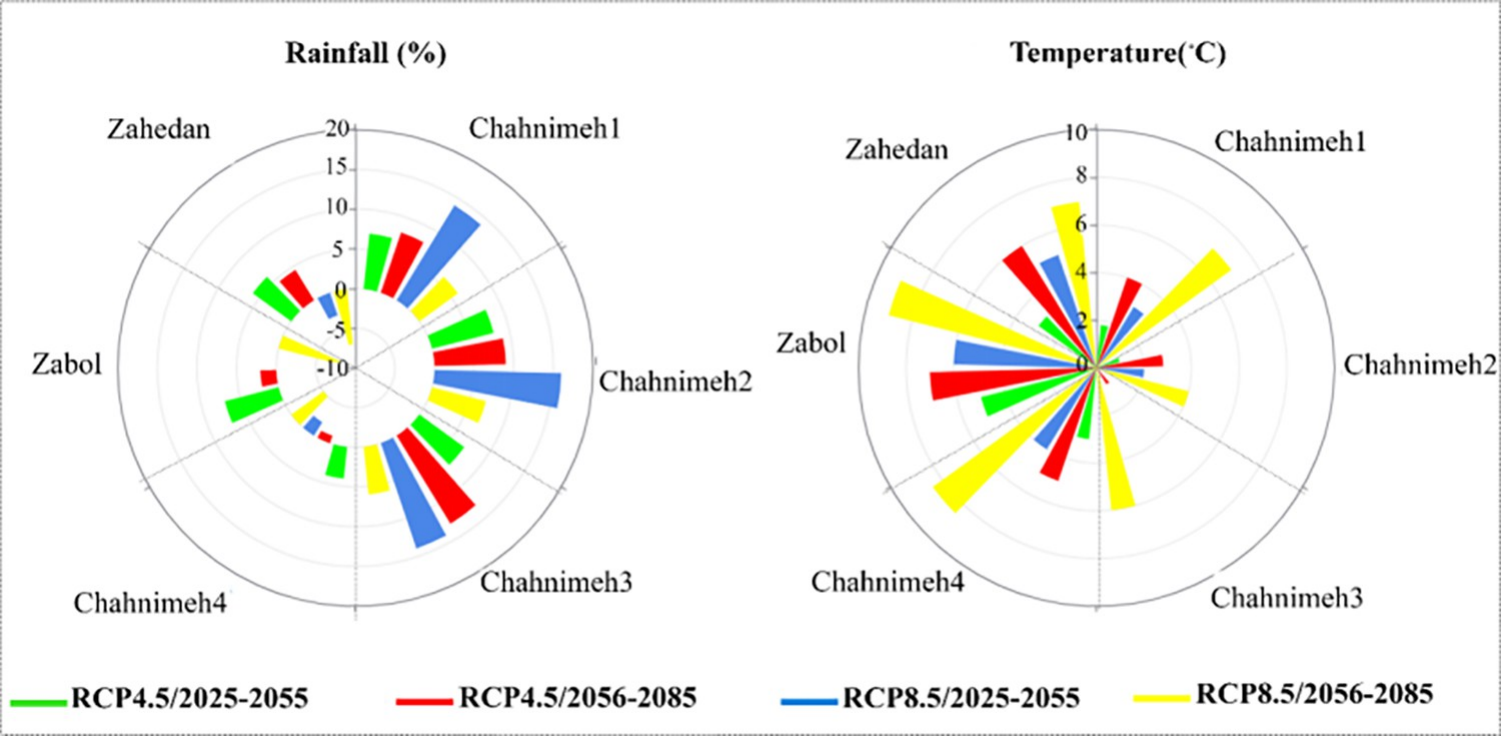
Rainfall and temperature adjustments under different scenarios.

Following the calibration and validation of the data, the projection of rainfall and temperature was conducted under the RCP4.5&RCP8.5 scenarios for the time periods (2025–2055&2056–2085). Based on the acquired results (Fig 6), the escalation in temperature and the decline in average rainfall are evident under the proposed scenarios. For instance, the difference in average rainfall between the RCP8.5(2025–2055) scenario and the RCP8.5(2056–2085) scenario exceeds -5%, thereby indicating a worsening water crisis in the region.

### Adopted optimal water supply to sub-sectors

Table 4 presents the optimal water allocation outputs across sectors for four scenarios: A1 = RCP4.5(2025–2055), A2 = RCP4.5(2056–2085), B1 = RCP8.5(2025–2055), and B2 = RCP8.5(2056–2085). Based on the findings, agricultural sector in Zabol received a greater share of water compared to the other sectors. Conversely, in Zahedan city, the industrial sector emerged as the primary beneficiary, receiving a substantial volume exceeding 40.00*10^6^*m*^3^. In addition, the domestic sector in Zahedan received nearly four times the amount of water allocated to Zabol. Notably, under scenario B2, this sector obtained the largest water allocation, amounting to 22.14*10^6^*m*^3^.

**Table 4:** Optimal water allocated to sectors (10^6^m^3^).

$x_{t}^{j}$		**A1**	**A2**	**B1**	**B2**
**Zabol**	**AGR**	47.36	48.17	49.74	50.53
	**IND**	10.13	09.72	10.67	11.58
	**DOM**	4.79	5.36	5.14	6.28
	**AGR**	19.76	21.08	19.39	22.53
**Zahedan**	**IND**	32.35	33.64	40.73	36.01
	**DOM**	18.19	20.04	21.36	22.14

Upon analyzing the system sustainability (Table 5), it becomes evident that the domestic sector attained the highest value in both sub-areas (0.491, 0.389). This signifies that the status of the domestic sector surpasses that of the other two sectors across all three factors: reliability, resilience (which remained unchanged as the system did not transition from failure to non-failure), and vulnerability.

**Table 5:** Analysis of sustainability measure.

	Reliability			Resilience			Vulnerability			Sustainability		
	AGR	IND	DOM	AGR	IND	DOM	AGR	IND	DOM	AGR	IND	DOM
**Zabol**	0.2	0.4	0.4	1	1	1	0.036	0.028	0.019	0.192	0.388	0.491
	DOM = IND>AGR			DOM = IND = AGR			AGR>IND>DOM			DOM>IND>AGR		
**Zahedan**	0.3	0.2	0.4	1	1	1	0.029	0.030	0.026	0.194	0.194	0.389
	DOM>AGR>IND			DOM = IND = AGR			IND>AGR>DOM			DOM>IND = AGR		

**Fig 7:**
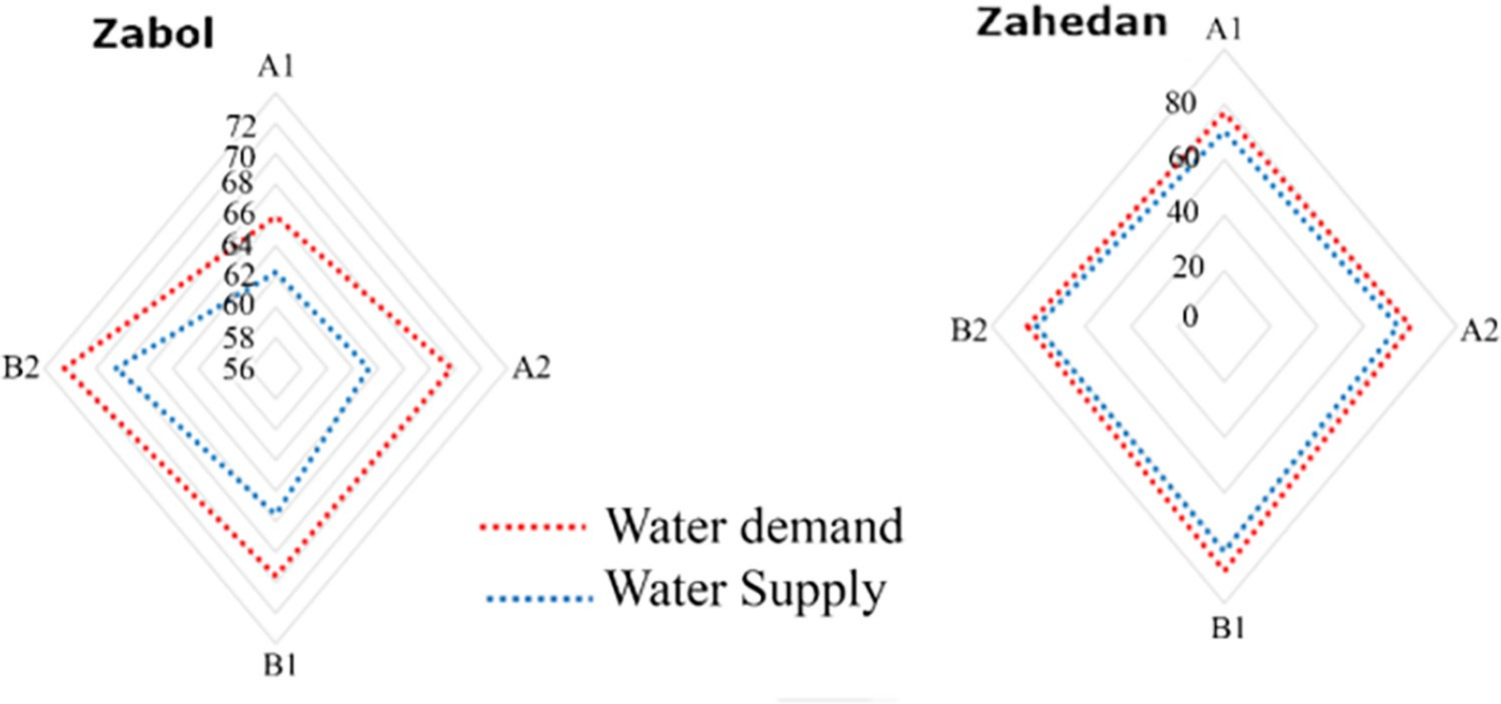
Maintaining the deviation between water demand and water supply.

In addition, the magnitude of deviation between water demand and water supply is depicted in Fig 7. The highest deviation was observed for Zahedan city under scenario B1, wherein the total supply amounted to 81.48*10^6^*m*^3^ and the total demand reached 88.32*10^6^*m*^3^. Similarly, under scenario A2, Zabol city exhibited a deviation with a total supply of 63.25*10^6^*m*^3^ and a total demand of 69.98*10^6^*m*^3^. Despite the reduction in the deviation between water demand and water supply achieved through the proposed optimal framework, the unsustainability of the water supply system remains evident due to the decline in the runoff rate.

### Adjusted strategies for analysis of sustainability measure under demand shrinkage scenarios

Recent studies [30, 33] have indicated that the Sistan Basin will encounter a decrease in rainfall and an increase in temperature, thereby exacerbating the scarcity of water resources. In addition, the growth in population contributes to the widening gap between water demand and water supply. However, prevailing adaptation measures for water supply primarily concentrate on proposing strategies for water allocation across various sectors, while neglecting the development of water demand management options. In light of this, it is necessary to put forth practical managerial insights that align with the current circumstances. These insights may involve the implementation of leverage policies and water tariffs, the utilization of advanced technologies such as drip irrigation systems, and the treatment of wastewater. Such measures can serve as viable alternatives for conserving water resources. Therefore, this study employed demand shrinkage scenarios (10% and 20% lower than the demand parameter specified in Table 1) to assess the sustainability of the water supply system.

According to the findings (presented in Table 6), the reliability and resilience of all sectors exhibited improvement when compared to their current status (Table 5). However, the vulnerability values for three sectors did not undergo significant changes. Overall, the system’s sustainability has been enhanced through the implementation of the demand shrinkage strategy.

**Table 6:** The impact of demand shrinkage scenarios on sustainability measure.

*j*	Demand shrinkage	Reliability			Resilience			Vulnerability			Sustainability		
		AGR	IND	DOM	AGR	IND	DOM	AGR	IND	DOM	AGR	IND	DOM
**Zabol**	10%	0.3	0.5	0.6	1	1	0.2	0.040	0.035	0.015	0.288	0.482	0.591
**Zahedan**		0.4	0.6	0.6	1	0.2	1	0.033	0.037	0.028	0.387	0.578	0.583
**Zabol**	20%	0.7	0.5	0.5	0.2	0.4	0.5	0.041	0.026	0.017	0.671	0.487	0.492
**Zahedan**		0.5	0.6	0.7	0.2	0.3	0.6	0.032	0.039	0.021	0.484	0.672	0.587

## Discussion

To enhance the sustainability of system, it is necessary to develop novel adaptation measures that prioritize water supply among key sectors, considering the varying water requirements of intersectoral users based on their respective priorities. Nevertheless, in light of the preceding analysis, several key considerations should be considered:

Highlight 1: The distribution patterns of population growth in the coming years should be analyzed to facilitate demand management.

Considering that the level of water consumption is contingent upon the rate of population growth [35], the primary driver behind increased water consumption in the irrigation system, is the demand for additional food production. In practical terms, accurately assessing the potential gap between water demand and supply in the future necessitates the prediction of trends based on population growth rates and other influential variables. In light of the timing and magnitude of this gap, it is incumbent upon local and national authorities to devise appropriate measures aimed at minimizing the gap and meeting the projected demand.

Highlight 2: The challenge of water supply in the irrigation system requires adaptation measures.

The agricultural sector, being the largest consumer of water, has been directly affected by the drought, which has disrupted its supply chain. In addition, the inefficient and suboptimal utilization of water in the irrigation system presents significant operational hurdles for other businesses in a region that heavily rely on surface waters.

However, conducting a comprehensive assessment of the potential impacts of climate change on the agricultural sector at a regional scale proves to be highly effective. Additionally, the implementation of novel strategies, such as smart irrigation technologies, will greatly contribute to the conservation of water resources.

Based on the aforementioned description, the proposed model exhibits the following advantages:

1. The proposed framework serves as a practical mechanism for optimizing water supply at the regional level, thereby enhancing sustainability across various scenarios and offering long-term perspectives for governing bodies.
2. To minimize the impacts of uncertain runoff on the optimization of water supply, it is crucial to implement suitable adaptation measures that ensure a minimal deviation between water supply and demand. Merely concentrating on water resource management alone does not yield swift resolutions to the prevailing challenges. Instead, the exploration of innovative strategies, such as the implementation of demand shrinkage scenarios, can foster enhanced long-term sustainability in the system.

## Conclusions

However, the challenge of meeting multi-sectoral water demand has emerged as a formidable task for authorities seeking to minimize failure and enhance the sustainability of the system. In light of this, a novel optimal framework is proposed in this study, which puts forth a refined definition for the sustainability of the water supply system. The primary objective of this framework is to reduce system failure and thereby enhance sustainability. Considering the influence of climate uncertainty on the supply process, the SWAT mechanism is employed as a supplementary tool to project climate data. In addition, due to the mismatch between water supply and water demand parameters, caused by both increased demand and the stress of climate change, an adaptive measure in the form of a demand shrinkage scenario is assumed. This scenario is analyzed to assess the impact of demand management on the sustainability of the system. The final output reveals that the domestic sector, characterized by the largest consumer base, exhibits the highest sensitivity to reliability and a lower vulnerability. Therefore, it exerts a significant influence on the improvement of system sustainability. The findings of this study highlight the importance of adopting a long-term perspective that focuses on optimizing the three key indicators of reliability, resilience, and vulnerability, as this approach leads to enhanced sustainability.

## Supporting information

S1 File (DOCX)

S1 Dataset (RAR)

## References

[1] Brown CM, Lund JR, Cai X, Reed PM, Zagona EA, Ostfeld A, et al. The future of water resources systems analysis: Toward a scientific framework for sustainable water management. Water resources research. 2015 Aug; 51(8):6110–24. 10.1002/2015WR017114.

[2] Xiong W, Li Y, Pfister S, Zhang W, Wang C, Wang P. Improving water ecosystem sustainability of urban water system by management strategies optimization. Journal of environmental management. 2020 Jan 15; 254:109766. doi: 10.1016/j.jenvman.2019.109766 31733479

[3] Xu Z, Yao L, Zhou X, Moudi M, Zhang L. Optimal irrigation for sustainable development considering water rights transaction: A Stackelberg-Nash-Cournot equilibrium model. Journal of Hydrology. 2019 Aug 1; 575:628–37. 10.1016/j.jhydrol.2019.05.063.

[4] Rathnayaka K, Malano H, Arora M. Assessment of sustainability of urban water supply and demand management options: a comprehensive approach. Water. 2016 Dec 15; 8(12):595. 10.3390/w8120595.

[5] Ajami NK, Hornberger GM, Sunding DL. Sustainable water resource management under hydrological uncertainty. Water Resources Research. 2008 Nov; 44(11). 10.1029/2007WR006736.

[6] Herman JD, Reed PM, Zeff HB, Characklis GW. How should robustness be defined for water systems planning under change?. Journal of Water Resources Planning and Management. 2015 Oct 1; 141(10):04015012. 10.1061/(ASCE)WR.1943-5452.0000509.

[7] Galaitsi SE, Keisler JM, Trump BD, Linkov I. The need to reconcile concepts that characterize systems facing threats. Risk Analysis. 2021 Jan; 41(1):3–15. doi: 10.1111/risa.13577 32818299

[8] Schaab DA, Weckmann S, Kuhlmann T, Sauer A. Simulative analysis of a flexible, robust and sustainable energy supply through industrial smart-dc-grid with distributed grid management. Procedia CIRP. 2018 Jan 1; 69:366–70. 10.1016/j.procir.2017.11.037.

[9] Gu W, Shao D, Tan X, Shu C, Wu Z. Simulation and optimization of multi-reservoir operation in inter-basin water transfer system. Water resources management. 2017 Sep; 31:3401–12. 10.1007/s11269-017-1675-9.

[10] Srdjevic Z, Srdjevic B. An extension of the sustainability index definition in water resources planning and management. Water Resources Management. 2017 Mar; 31:1695–712. 10.1007/s11269-017-1609-6.

[11] Afshar A, Khosravi M, Molajou A. Assessing adaptability of cyclic and non-cyclic approach to conjunctive use of groundwater and surface water for sustainable management plans under climate change. Water Resources Management. 2021 Sep; 35(11):3463–79. 10.1007/s11269-021-02887-3.

[12] Butler D, Ward S, Sweetapple C, Astaraie‐Imani M, Diao K, Farmani R, et al. Reliable, resilient and sustainable water management: the Safe & SuRe approach. Global Challenges. 2017 Jan; 1(1):63–77. 10.1002/gch2.1010. 31565260 PMC6655362

[13] Abdi-Dehkordi M, Bozorg-Haddad O, Chu X. Development of a combined index to evaluate sustainability of water resources systems. Water Resources Management. 2021 Jul; 35(9):2965–85. 10.1007/s11269-021-02880-w.

[14] Kotir JH, Smith C, Brown G, Marshall N, Johnstone R. A system dynamics simulation model for sustainable water resources management and agricultural development in the Volta River Basin, Ghana. Science of the Total Environment. 2016 Dec 15; 573:444–57. doi: 10.1016/j.scitotenv.2016.08.081 27572537

[15] Chen Y, He L, Lu H, Li J, Ren L. Planning for regional water system sustainability through water resources security assessment under uncertainties. Water Resources Management. 2018 Jul; 32:3135–53. 10.1007/s11269-018-1981-x.

[16] Arnell NW, Gosling SN. The impacts of climate change on river flow regimes at the global scale. Journal of Hydrology. 2013 Apr 12; 486:351–64. 10.1016/j.jhydrol.2013.02.010.

[17] Zhou J, He D, Xie Y, Liu Y, Yang Y, Sheng H, et al. Integrated SWAT model and statistical downscaling for estimating streamflow response to climate change in the Lake Dianchi watershed, China. Stochastic Environmental Research and Risk Assessment. 2015 May; 29:1193–210. 10.1007/s00477-015-1037-1.

[18] Fechete F, Nedelcu A. Performance management assessment model for sustainable development. Sustainability. 2019 May 15; 11(10):2779. 10.3390/su11102779.

[19] Zhang C, Xu B, Li Y, Fu G. Exploring the relationships among reliability, resilience, and vulnerability of water supply using many-objective analysis. Journal of water resources planning and management. 2017 Aug 1; 143(8):04017044. 10.1061/(ASCE)WR.1943-5452.0000787.

[20] Hazbavi Z, Baartman JE, Nunes JP, Keesstra SD, Sadeghi SH. Changeability of reliability, resilience and vulnerability indicators with respect to drought patterns. Ecological Indicators. 2018 Apr 1; 87:196–208. 10.1016/j.ecolind.2017.12.054.

[21] Ren K, Huang S, Huang Q, Wang H, Leng G, Fang W, et al. Assessing the reliability, resilience and vulnerability of water supply system under multiple uncertain sources. Journal of Cleaner Production. 2020 Apr 10; 252:119806. 10.1016/j.jclepro.2019.119806.

[22] Asefa T, Clayton J, Adams A, Anderson D. Performance evaluation of a water resources system under varying climatic conditions: Reliability, Resilience, Vulnerability and beyond. Journal of Hydrology. 2014 Jan 16; 508:53–65. 10.1016/j.jhydrol.2013.10.043.

[23] Nassour J, Leykin D, Elhadad M, Cohen O. Computational Text Analysis of A Scientific Resilience Management Corpus: Environmental Insights and Implications. Journal of Environmental Informatics. 2020 Sep 1; 36(1). 10.3808/jei.201900423.

[24] Kim S, Jun HD, Yoo DG, Kim JH. A framework for improving reliability of water distribution systems based on a segment-based minimum cut-set approach. Water. 2019 Jul 23; 11(7):1524. 10.3390/w11071524.

[25] Maestro T, Nicolosi V, Cancelliere A, Bielza M. Impacts of climate change, hydrological drought mitigation measures and irrigation demand on water supply system performance. Eur Water. 2014; 45(46):25–33.

[26] Goharian E, Burian SJ, Lillywhite J, Hile R. Vulnerability assessment to support integrated water resources management of metropolitan water supply systems. Journal of Water Resources Planning and Management. 2017 Mar 1; 143(3):04016080. 10.1061/(ASCE)WR.1943-5452.0000738.

[27] Karamouz M, Mohammadpour P, Mahmoodzadeh D. Assessment of sustainability in water supply-demand considering uncertainties. Water Resources Management. 2017 Sep; 31:3761–78. 10.1007/s11269-017-1703-9.

[28] Ghaffari Moghadam Z, HashemiTabar M, Sardar Shahraki A. Economic Model for Optimal Allocation of Water Resources with an Emphasis on Risk and Consistency Index in the Sistan Region: The Application of Interval Two-Stage Stochastic Programming Method. Environmental Energy and Economic Research. 2022 Aug 1; 6(3):1–3. 10.22097/EEER.2022.321052.1235

[29] Farrokhzadeh S, Hashemi Monfared SA, Azizyan G, Sardar Shahraki A, Ertsen MW, Abraham E. Sustainable water resources management in an arid area using a coupled optimization-simulation modeling. Water. 2020 Mar 21; 12(3):885. 10.3390/w12030885.

[30] He Y, Mahdi M, Huang P, Xie G, Galoie M, Shafi M. Investigation of climate change adaptation impacts on optimization of water allocation using a coupled SWAT-bi level programming model. Wetlands. 2021 Mar; 41:1–8. 10.1007/s13157-021-01434-5.

[31] Shahraki M, Mahmudy Gharaie MH, Moussavi-Harami R, Rashki A. Geochemistry of Bandan River sediments in Sistan Basin (Eastern Iran): implication for provenance and environmental impact on the Hamoun Lake pollution. Environmental earth sciences. 2021 Jan; 80:1–7. 10.1007/s12665-020-09313-8.

[32] Mianabadi H, Alioghli S, Morid S. Quantitative evaluation of ‘No-harm’rule in international transboundary water law in the Helmand River basin. Journal of Hydrology. 2021 Aug 1; 599:126368. 10.1016/j.jhydrol.2021.126368.

[33] Yao L, Xu Z, Moudi M, Li Z. Optimal water allocation in Iran: a dynamic bi-level programming model. Water Supply. 2019 Jun 1; 19(4):1120–8. 10.2166/ws.2018.165.

[34] Mahdi M, Xueqian S, Gai Q, Basirialmahjough M, Yuan H. Improving robustness of water supply system using a multi-objective robust optimization framework. Environmental Research. 2023 May 29:116270. doi: 10.1016/j.envres.2023.116270 37257741

[35] Boretti A, Rosa L. Reassessing the projections of the world water development report. NPJ Clean Water. 2019 Jul 31; 2(1):15. 10.1038/s41545-019-0039-9.

[36] Liu D, Guo S, Shao Q, Liu P, Xiong L, Wang L, et al. Assessing the effects of adaptation measures on optimal water resources allocation under varied water availability conditions. Journal of Hydrology. 2018 Jan 1; 556:759–74. 10.1016/j.jhydrol.2017.12.002.

[37] Themeßl MJ, Gobiet A, Heinrich G. Empirical-statistical downscaling and error correction of regional climate models and its impact on the climate change signal. Climatic Change. 2012 May; 112:449–68. 10.1007/s10584-011-0224-4

