# Supplementary material for: A novel objective for improving the sustainability of water supply system regarding hydrological response
Source: PLoS One. 2023 Nov 30;18(11):e0294578. doi: 10.1371/journal.pone.0294578 (PMC10688848; doi:10.1371/journal.pone.0294578)
Supplement: S1 File — (DOCX) [file pone.0294578.s001.docx]

library(Rdonlp2)

I= data.frame(array(c(rep(5,10)),c(1,10)))

DEM=data.frame(array(c(1.02,3.67,3.71,0.89,2.57,3.59,1.30,

4.63,3.65,1.56,4.68,3.76,1.00,4.80,3.84,1.34,

3.77,3.84,1.00,3.75,3.84,1.45,4.91,3.88,0.98,

4.76,4.10,1.65,4.61,3.68),c(3,10)))

sum_DEM = rep(0,10)

for(i in 1:10)

{sum_DEM[i] = sum(DEM[,i])}

DEM_series= c()

for(i in 1:10){

for(k in 1:3){

DEM_series = c(DEM_series,DEM[k,i])

}

}

fn = function(t){

s = t[1:10]

SUP = t[11:40]

s = data.frame(array(s,c(1,10)))

SUP = data.frame(array(SUP,c(3,10)))

s[1,1]=1.4+5.0-sum(SUP[,1])

for(i in 2:10){

s[1,i]=s[1,i-1]+I[1,i-1]-sum(SUP[,i-1])

}

z=data.frame(matrix(nrow=3,ncol=10))

for(k in 1:3){

for(i in 1:10){

if(SUP[k,i]<DEM[k,i]){

z[k,i]=1

}

else {z[k,i]=0}

}

}

a = data.frame(matrix(nrow=3,ncol=1))

for(k in 1:3){

a[k,1]= (10- sum(z[k,]))/10

}

w=data.frame(matrix(nrow=3,ncol=10))

w[,10]=0

z[,11]=0

for(k in 1:3){

for(i in 1:10){

if(z[k,i]==1&z[k,i+1]==0){

w[k,i]=1

}

else {w[k,i]=0}

}

}

beta = data.frame(matrix(nrow=3,ncol=1))

for(k in 1:3){

beta[k,1]=sum(w[k,])/(11-sum(z[k,]))

}

SH=data.frame(matrix(nrow=3,ncol=10))

for(k in 1:3){

for(i in 1:10){

if(SUP[k,i]>DEM[k,i]||SUP[k,i]==DEM[k,i]){

SH[k,i]=0

}

else {SH[k,i]=DEM[k,i]-SUP[k,i]}

}

}

r = data.frame(matrix(nrow=3,ncol=1))

for(k in 1:3){

r[k,1]=(1/sum(z[k,]))*(sum(SH[k,])/sum(DEM[k,]))

}

result = 0

for(k in 1:3){

result = result+(1-a[k,1])+(1-beta[k,1])+r[k,1]

}

return (result)

}

par.l = c(rep(1.4,10), rep(0,30)); par.u = c(rep(8.6,10), rep(Inf,30))

A = matrix(c(rep(0,10),rep(1,3),rep(0,27),

rep(0,10),rep(0,3),rep(1,3),rep(0,24),

rep(0,10),rep(0,6),rep(1,3),rep(0,21),

rep(0,10),rep(0,9),rep(1,3),rep(0,18),

rep(0,10),rep(0,12),rep(1,3),rep(0,15),

rep(0,10),rep(0,15),rep(1,3),rep(0,12),

rep(0,10),rep(0,18),rep(1,3),rep(0,9),

rep(0,10),rep(0,21),rep(1,3),rep(0,6),

rep(0,10),rep(0,24),rep(1,3),rep(0,3),

rep(0,10),rep(0,27),rep(1,3),

rep(0,10),rep(1,1),rep(0,29),

rep(0,10),rep(0,1),rep(1,1),rep(0,28),

rep(0,10),rep(0,2),rep(1,1),rep(0,27),

rep(0,10),rep(0,3),rep(1,1),rep(0,26),

rep(0,10),rep(0,4),rep(1,1),rep(0,25),

rep(0,10),rep(0,5),rep(1,1),rep(0,24),

rep(0,10),rep(0,6),rep(1,1),rep(0,23),

rep(0,10),rep(0,7),rep(1,1),rep(0,22),

rep(0,10),rep(0,8),rep(1,1),rep(0,21),

rep(0,10),rep(0,9),rep(1,1),rep(0,20),

rep(0,10),rep(0,10),rep(1,1),rep(0,19),

rep(0,10),rep(0,11),rep(1,1),rep(0,18),

rep(0,10),rep(0,12),rep(1,1),rep(0,17),

rep(0,10),rep(0,13),rep(1,1),rep(0,16),

rep(0,10),rep(0,14),rep(1,1),rep(0,15),

rep(0,10),rep(0,15),rep(1,1),rep(0,14),

rep(0,10),rep(0,16),rep(1,1),rep(0,13),

rep(0,10),rep(0,17),rep(1,1),rep(0,12),

rep(0,10),rep(0,18),rep(1,1),rep(0,11),

rep(0,10),rep(0,19),rep(1,1),rep(0,10),

rep(0,10),rep(0,20),rep(1,1),rep(0,9),

rep(0,10),rep(0,21),rep(1,1),rep(0,8),

rep(0,10),rep(0,22),rep(1,1),rep(0,7),

rep(0,10),rep(0,23),rep(1,1),rep(0,6),

rep(0,10),rep(0,24),rep(1,1),rep(0,5),

rep(0,10),rep(0,25),rep(1,1),rep(0,4),

rep(0,10),rep(0,26),rep(1,1),rep(0,3),

rep(0,10),rep(0,27),rep(1,1),rep(0,2),

rep(0,10),rep(0,28),rep(1,1),rep(0,1),

rep(0,10),rep(0,29),rep(1,1)

)

,40,byrow=TRUE)

lin.l = rep(0,40); lin.u = c(sum_DEM,DEM_series)

nlcon1 = function(t){

s = t[1:10]

SUP = t[11:40]

s = data.frame(array(s,c(1,10)))

SUP = data.frame(array(SUP,c(3,10)))

sum(SUP[,1])/(1.4+I[1,1])

}

nlcon2 = function(t){

s = t[1:10]

SUP = t[11:40]

s = data.frame(array(s,c(1,10)))

SUP = data.frame(array(SUP,c(3,10)))

sum(SUP[,2])/(s[1,1]+I[1,2])

}

nlcon3 = function(t){

s = t[1:10]

SUP = t[11:40]

s = data.frame(array(s,c(1,10)))

SUP = data.frame(array(SUP,c(3,10)))

sum(SUP[,3])/(s[1,2]+I[1,3])

}

nlcon4 = function(t){

s = t[1:10]

SUP = t[11:40]

s = data.frame(array(s,c(1,10)))

SUP = data.frame(array(SUP,c(3,10)))

sum(SUP[,4])/(s[1,3]+I[1,4])

}

nlcon5 = function(t){

s = t[1:10]

SUP = t[11:40]

s = data.frame(array(s,c(1,10)))

SUP = data.frame(array(SUP,c(3,10)))

sum(SUP[,5])/(s[1,4]+I[1,5])

}

nlcon6 = function(t){

s = t[1:10]

SUP = t[11:40]

s = data.frame(array(s,c(1,10)))

SUP = data.frame(array(SUP,c(3,10)))

sum(SUP[,6])/(s[1,5]+I[1,6])

}

nlcon7 = function(t){

s = t[1:10]

SUP = t[11:40]

s = data.frame(array(s,c(1,10)))

SUP = data.frame(array(SUP,c(3,10)))

sum(SUP[,7])/(s[1,6]+I[1,7])

}

nlcon8 = function(t){

s = t[1:10]

SUP = t[11:40]

s = data.frame(array(s,c(1,10)))

SUP = data.frame(array(SUP,c(3,10)))

sum(SUP[,8])/(s[1,7]+I[1,8])

}

nlcon9 = function(t){

s = t[1:10]

SUP = t[11:40]

s = data.frame(array(s,c(1,10)))

SUP = data.frame(array(SUP,c(3,10)))

sum(SUP[,9])/(s[1,8]+I[1,9])

}

nlcon10 = function(t){

s = t[1:10]

SUP = t[11:40]

s = data.frame(array(s,c(1,10)))

SUP = data.frame(array(SUP,c(3,10)))

sum(SUP[,10])/(s[1,9]+I[1,10])

}

nlin.l = c(rep(0,10)); nlin.u = c(rep(1,10))

p =c(rep(2,10),

0.9,2.57,3.00,

0.89,2.57,3.09,

1.10,3.93,3.65,

1.16,3.68,3.76,

0.90,4.50,3.84,

1.14,3.57,3.84,

0.80,3.45,4.04,

0.95,4.51,3.88,

0.98,4.56,3.30,

0.95,2.9,3.68)

ret = donlp2(p, fn, par.u=par.u, par.l=par.l, A, lin.l=lin.l,lin.u=lin.u,

nlin = list(nlcon1,nlcon2,nlcon3,nlcon4,nlcon5,nlcon6,nlcon7,

nlcon8,nlcon9,nlcon10),

nlin.u=nlin.u, nlin.l=nlin.l)

ret$par

#result = fn(rep(50,40))

#############################################################################

t = ret$par

s = t[1:10]

SUP = t[11:40]

s = data.frame(array(s,c(1,10)))

SUP = data.frame(array(SUP,c(3,10)))

z=data.frame(matrix(nrow=3,ncol=10))

for(k in 1:3){

for(i in 1:10){

if(SUP[k,i]<DEM[k,i]){

z[k,i]=1

}

else {z[k,i]=0}

}

}

a = data.frame(matrix(nrow=3,ncol=1))

for(k in 1:3){

a[k,1]= (10- sum(z[k,]))/10

}

w=data.frame(matrix(nrow=3,ncol=10))

w[,10]=0

z[,11]=0

for(k in 1:3){

for(i in 1:10){

if(z[k,i]==1&z[k,i+1]==0){

w[k,i]=1

}

else {w[k,i]=0}

}

}

beta = data.frame(matrix(nrow=3,ncol=1))

for(k in 1:3){

beta[k,1]=sum(w[k,])/(11-sum(z[k,]))

}

SH=data.frame(matrix(nrow=3,ncol=10))

for(k in 1:3){

for(i in 1:10){

if(SUP[k,i]>DEM[k,i]||SUP[k,i]==DEM[k,i]){

SH[k,i]=0

}

else {SH[k,i]=DEM[k,i]-SUP[k,i]}

}

}

r = data.frame(matrix(nrow=3,ncol=1))

for(k in 1:3){

r[k,1]=(1/sum(z[k,]))*(sum(SH[k,])/sum(DEM[k,]))

}

result = 0

for(k in 1:3){

result = result+(1-a[k,1])+(1-beta[k,1])+r[k,1]

}

sum_SUP = rep(0,10)

for(i in 1:10)

{sum_SUP[i] = sum(SUP[,i])}
